# Steady-State Visual Evoked Potentials Elicited from Early Visual Cortex Reflect Both Perceptual Color Space and Cone-Opponent Mechanisms

**DOI:** 10.1093/texcom/tgaa059

**Published:** 2020-09-01

**Authors:** Sae Kaneko, Ichiro Kuriki, Søren K Andersen

**Affiliations:** Frontier Research Institute for Interdisciplinary Sciences, Tohoku University; Aramaki aza Aoba 6-3, Aoba-ku, Sendai, Miyagi, 980-8578, Japan; Research Institute of Electrical Communication, Tohoku University, 2-1-1 Katahira, Aoba-ku, Sendai, Miyagi, 980-8577, Japan; Research Institute of Electrical Communication, Tohoku University, 2-1-1 Katahira, Aoba-ku, Sendai, Miyagi, 980-8577, Japan; School of Psychology, University of Aberdeen, William Guild Building, Aberdeen, AB24 3UB, UK

**Keywords:** color representation, EEG, intermediate hues, isoluminant colors, primary visual cortex

## Abstract

Colors are represented in the cone-opponent signals, L-M versus S cones, at least up to the level of inputs to the primary visual cortex. We explored the hue selective responses in early cortical visual areas through recordings of steady-state visual evoked potentials (SSVEPs), elicited by a flickering checkerboard whose color smoothly swept around the hue circle defined in a cone-opponent color space. If cone opponency dominates hue representation in the source of SSVEP signals, SSVEP amplitudes as a function of hue should form a profile that is line-symmetric along the cardinal axes of the cone-opponent color space. Observed SSVEP responses were clearly chromatic ones with increased SSVEP amplitudes and reduced response latencies for higher contrast conditions. The overall elliptic amplitude profile was significantly tilted away from the cardinal axes to have the highest amplitudes in the “lime-magenta” direction, indicating that the hue representation in question is not dominated by cone-opponency. The observed SSVEP amplitude hue profile was better described as a summation of a perceptual response and cone-opponent responses with a larger weight to the former. These results indicate that hue representations in the early visual cortex, measured by the SSVEP technique, are possibly related to perceptual color contrast.

## Introduction

Any color we see starts with the excitation of 3 types of cones at the retina. These cones have different peak sensitivity wavelength and we call them long-, medium-, and short-wavelength sensitive (in short, L-, M-, and S-) cones. The signals from these cones are transformed via neurons in the retina that finally form the color selectivity of ganglion cells. Color selective retinal ganglion cells receive opposed L- and M- cone signals or S cone signals opposed to the sum of L- and M-cone signals, and these signals are further carried into the lateral geniculate nucleus (LGN) (Derrington et al. 1984; Hanazawa et al. 2000). This means 2 cone-opponent signals, L versus M cone signals and S versus L + M signals, constitute the color representation in the retinal ganglion cells and LGN.

However, how and where these cone-opponent signals are reorganized after LGN to represent millions of colors we perceive is an unsolved question. The main question we address here is whether the hue representation in LGN is carried on into the early visual cortex as well, or the hue information starts to be reconstructed in a more complex fashion. More specifically, we intend to explore how “intermediate” hues, that is, the hues that excite both types of cone-opponent neurons in LGN, are represented in the area. Previous single-unit studies on the macaque brains have shown that there are neurons that selectively respond to intermediate hues as early as the primary visual cortex (V1) (Lennie et al. 1990; Hanazawa et al. 2000; Wachtler et al. 2003; Hass and Horwitz 2013). Although selectivity to intermediate hues is achievable by the combination of cone-opponent signals in the cortex (Hanazawa et al. 2000; Wachtler et al. 2003), whether such reconstructions of the signals are carried out in human V1 or other early visual areas is still an unsolved issue.

Noncone opponent representation of colors in human V1 has been suggested by some previous functional magnetic resonance imaging (fMRI) studies. For example, Goddard et al. (2010) showed that the blood oxygenation level dependent (BOLD; Ogawa et al. 1992) responses in human V1 evoked by hue modulation along the “lime (−L + M, –S)-magenta (+L–M, +S)” and “orange (+L–M, –S)-cyan (−L + M, +S)” directions could be successfully discriminated by multivariate pattern analysis (MVPA). Another interesting feature found in this study was that the lime-magenta stimulus elicited a stronger BOLD response, on average, than the hue modulation along the orange-cyan direction. Theoretically, both modulations should invoke the same level of excitation in the cone-opponent neurons or the linear combinations of outputs of these neurons, since both color pairs are carefully designed to deliver the same extent of stimulation in cone-opponent mechanisms. Therefore, the observed difference between the 2 conditions suggests that cone signals are combined nonlinearly in V1. Other studies using MVPA of the BOLD response also found that although neurons in V1 respond strongly to the cardinal hues, they cluster according to perceptually defined hues (such as unique hues), not cardinal hues (Parkes et al. 2009; Kuriki et al. 2011). These studies have “indirectly” succeeded in demonstrating the presence of neurons selective to the intermediate directions of cone-opponent space, which had been suggested by psychophysical studies for several decades (Krauskopf et al. 1986; Webster and Mollon 1991). However, this evidence is still “indirect,” because it did not reveal the detailed nature, such as population histogram, of neurons selective to intermediate colors in the cone-opponent space.

Other studies suggest that the cone-opponent representation is inherited in V1. For example, Engel et al. (1997) demonstrated that the responses from human V1 and V2 are mostly cone-opponent. Another MVPA study in fMRI also suggests cone-opponent representation in V1, unlike V4 where colors are represented in a way that is more relevant to perceived colors (Brouwer and Heeger 2009, 2013). This is unsurprising because cone-opponent signals are fed into the input layer of V1 from LGN parvo- and konio-cellular neurons. The key question is whether these cone-opponent signals still dominate the color representation in the early stages of the visual cortex, which is yet to be answered.

Recently, Kuriki et al. (2015) demonstrated that intermediate hues are represented independently from the cardinal hues in wide areas of the human visual cortex, namely V1 to V4. They found that a significant number of voxels in these areas showed selectivity to intermediate hues. They also showed through an adaptation experiment that these selective responses to noncardinal hues are not the responses of the cardinal mechanisms. This study, to our knowledge, is the first direct evidence of intermediate hue representations in human V1, which supports what was discovered in the brains of other primates by previous physiological studies (Hanazawa et al. 2000; Wachtler et al. 2003). Histograms of the hue selectivity of the voxel population showed strong anisotropy along the hue circle. However, it was not clear from this study how large this anisotropy was in proportion to the overall chromatic responses since the experimental design was intended to exclude voxels with bilateral hue selectivity (Kuriki et al. 2015).

In the present study, we measured the electrophysiological responses to various hues defined by a cone-opponent color space, using steady-state visual evoked potentials (SSVEPs). We designed our stimuli similarly to the ones used in Kuriki et al. (2015) to complement and expand their findings using the different modality of measurement. The use of SSVEP can have several advantages over fMRI. First, the electrophysiological signal has much higher temporal resolution compared with BOLD signal, and therefore SSVEP technique allows us to analyze the response latency to different hues, as well as the response amplitude. In fact, the cycle length of 24 s in Kuriki et al. (2015) was chosen to maximize the efficiency of BOLD signal temporal resolution which takes 6 s in latency for null-to-peak, on average. Therefore, SSVEP amplitude modulation has a possibility to exhibit much finer modulation along a hue circle. Second, electrophysiological signals reflect the underlying neural mechanisms more directly than the BOLD signal (for BOLD signal origin see Kim and Ogawa 2012). Furthermore, the methods of Kuriki et al. (2015) were specifically designed to emphasize the activity of mechanisms with unidirectional hue-selectivity. Due to the process to exclude voxels that contained approximately equal number of neurons selective to opposite hues, their result lacks baseline hue-selective responses that could have been more uniform across hues. For example, voxels that contained both +L–M selective and –L + M selective neurons were excluded, and this could have caused the absence in population along the L–M axis in their results. Hence, it was not possible to assess the size of the differences relative to the overall activity to the hues. In other words, the resulting histogram in Kuriki et al. (2015) may be “the tip of an iceberg” of the actual population of hue-selective neurons, with more uniform hue selective populations as the “submerged” part. By comparison, SSVEP amplitudes are naturally measured on a ratio scale, that is, an amplitude of zero corresponds to no signal power at the respective frequency. Finally, the previous studies show the primary source of the SSVEP response is V1 (Di Russo et al. 2007) with contributions from other early visual areas such as V2 (Kim et al. 2007), which encouraged the use of SSVEPs for our current purpose (for the further discussion of the source of SSVEP signals, see section Source of the SSVEP Signals).

## Materials and Methods

### Participants

Eighteen adults (7 females, average age 26.9 years; including 2 of the authors) participated in this experiment. Two participants out of these 18 were excluded from the analyses because of their weak SSVEP amplitude (<0.3 μV) and poor signal-to-noise ratio (<4) for the lowest chromatic contrast condition. The remaining 16 participants consisted of 7 females with an average age of 27.7 years. All had normal or corrected-to-normal visual acuity and normal color vision tested with Ishihara pseudo-isochromatic plates. All participants gave written informed consent before their participation. The experimental procedure was approved by the ethics committee of the Research Institute of Electrical Communication, Tohoku University. This research was conducted in accordance with the Code of Ethics of the World Medical Association (Declaration of Helsinki).

### Apparatus

Stimuli were presented on a 21-in. Cathode-ray tube (CRT) monitor (SONY CPD-G520; Sony Corporation, Tokyo, Japan; 800 × 600 pixels, refresh rate 100 Hz) through a ViSaGe system (Cambridge Research Systems, Rochester, UK) controlled by a computer (DELL Precision T3500; Dell, Texas, USA). The trigger pulse for stimulus onset was sent via the ViSaGe system to synchronize visual stimulus presentation and electroencephalogram (EEG) recording. The monitor was calibrated with an OptiCal photometer (Cambridge Research Systems, Rochester, UK). Viewing distance was approximately 114 cm (binocular viewing). No chin rest was used in this experiment. Experiments were done in a dark room.

### Stimuli

#### Color Space

**Figure 1:**
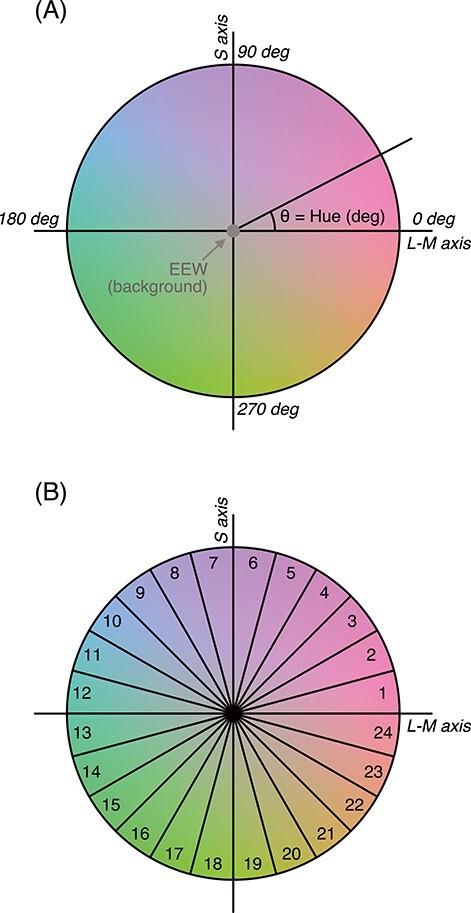
(*A*) Cone contrast color space used in the study. All hues in this study are described by their azimuth *θ*, or hue angle, on this color space. 0 deg corresponds to +L (“red”), and 90 deg corresponds to +S (“lavender”) contrast from the origin, EEW. (*B*) Hue sectors. Each sector spans 15 deg of hue angles, which corresponds to 1 s stimulation. Background colors show an approximation of the actual colors.

Following Kuriki et al. (2015), the stimulus colors used in this study were specified in a cone-opponent color space (Fig. 1*A*; MacLeod and Boynton 1979; Derrington et al. 1984). The origin of this color space was an equal energy white (EEW) at 30 cd/m^2^ (*x* = 0.33, *y* = 0.33). EEW was the reference point and was used for the background. The 2 cardinal axes either differentially vary L-cone/M-cone excitations or S-cone excitation variation with respect to the reference point. The former axis is called “L-M axis” and the latter “S axis” for short, hereafter. Cone excitations were calculated based on the cone fundamentals by Smith and Pokorny (1975). Cone contrast was defined as ratios between the increment of cone excitations from background gray with respect to the cone excitations for the background in L- and S-cone responses; that is, ΔL/L_w_ and ΔS/S_w_ (see Kuriki et al. 2015).

Cone contrasts along the L-M axis and S axis were maintained at a ratio of 1:10, which gives roughly equal multiples of detection thresholds for color changes along L-M and S axes, following Kuriki et al. (2015). Since the angular expression of hue strongly depends on the scaling between L-M and S axes, the scaling of these 2 axes is a very important issue. As it is known that color stimuli in either of the cardinal axes equated in cone contrasts ΔL/L_w_ and ΔS/S_w_ do not yield equal response in fMRI (Mullen et al. 2007), it was important to find the equilibrium point of the scaling between amplitudes in ΔL/L_w_ and ΔS/S_w_. The previous study by Kuriki et al. (2015) defined this scaling by brain activity and used a ratio of 1:5 for the recording of formal data and plotted the results in a space with a ratio of 1:10 (Figs 4 and 5 in Kuriki et al. 2015). The ratio of 1:10 was also employed in an fMRI decoding study by Kuriki et al. (2011) to demonstrate the ability to decode fMRI responses to color pairs of 225–45 deg (“lime-magenta”) versus 315–135 deg (“orange-cyan”). Therefore, we can maintain consistency in terms of hue angle representation across these studies by using the ratio of 1:10. In addition, our preliminary measurement showed no significant loss or gain of signal intensity along the S-cone axis, and therefore, we used the ratio of 1:10 in this study.

Hereafter, any hue in this color space is described by its azimuth, or hue angle, defined in this cone-contrast space. We defined L cone increment direction (red) as 0 deg and S cone increment direction as 90 deg (Fig. 1*A*). Before the EEG recording, each participant went through procedures for heterochromatic flicker photometry to make a necessary luminance adjustment along the L-M axis. Each participant underwent 10 repetitions of flicker photometry between “red” and EEW. The relative contribution of M-cone to the luminance channel (ϕ for *L* + ϕ*M* = *V*(λ); Eisner and MacLeod 1980; Ahn and MacLeod 1993; Kuriki and MacLeod 1998) was derived from the photometric value of the 2 probe colors for further calculations of stimulus chromaticity.

#### Checker Pattern

**Figure 2:**
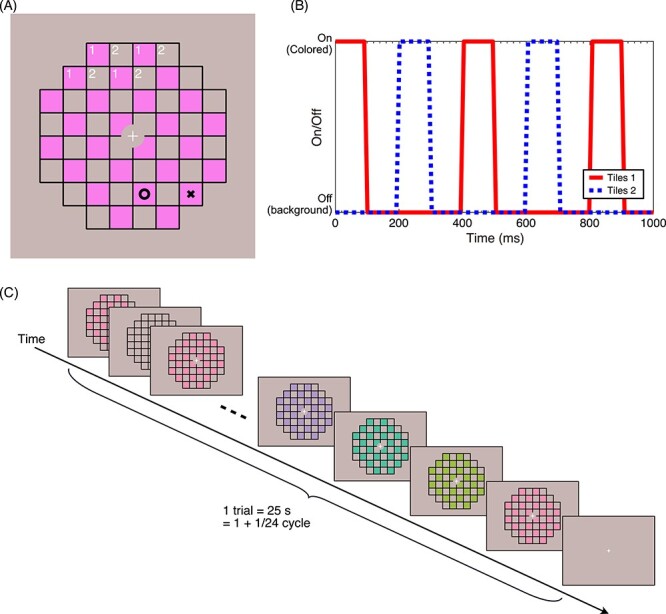
(*A*) Checkerboard pattern stimulus. O or X appeared at random timing at one of the colored tiles and participants were asked to press a button on seeing O but not X. O and X are shown here for the purpose of explanation and they were never shown simultaneously. Thin black grid was always visible during a trial. (*B*) Temporal on (colored)/off (background) pattern of the checkerboard. A trial always started with the on phase. “On” tiles switched between 2 groups at every “on” phase. (*C*) Schematic illustration of a trial. Stimulus hue was smoothly sweeping around the hue circle at 24 s/cycle rate. For a demonstrative movie of the stimulus, please refer to Supplementary Material.

We generated and presented the stimuli on MATLAB using the software libraries CRStoolbox (Cambridge Research Systems, Rochester, UK) and Psychtoolbox (Brainard 1997; Pelli 1997). A checkerboard pattern was presented on a gray (EEW) background (Fig. 2*A*). Each tile of the checkerboard was 0.76 × 0.76 deg, and the whole pattern subtended 6.10 × 6.10 deg. A small white fixation cross (0.33 × 0.33 deg) was shown at the center of the stimulus on a circular gray area. At any ON phase, half of the tiles were filled with an isoluminant chromatic color whereas the other tiles were filled with the gray background color. One on phase lasted 100 ms, followed by 100 ms off phase, in which the whole checkerboard was filled with the background color. This yielded a 5 Hz flicker rate as a result (Fig. 2*B*).

The color of the tiles was continuously and smoothly changing at the rate of 24 s/cycle (24 s of the presentation included all possible hues in the color space in Fig. 1*A*; see Fig. 2*C* and a movie in Supplementary Material). The hue change started at the edge of one of the 24 hue sectors (see Fig. 1*B*, counterbalanced). We had 2 directions of the hue change, clockwise or counterclockwise along the hue circle in Fig. 1*A*, in order to cancel out the potential effect of the progressive adaptation and/or the response temporal lag. Each ON phase presented a slightly different static hue even within a hue sector. Within a single hue sector (15 deg), there were 5 “on” phases, which means each “on” phase hue is advanced by 3 deg from the previous one (during the “off” phase when no tile was colored, there was no advancement in hue).

There were 3 chromatic contrast conditions: “full,” “half” and “quarter.” For “full” condition, colors were chosen from the maximum-cone contrast hue circle (maximum L cone contrast 8%, maximum S cone contrast 80%) available on the CRT monitor. For “half” condition, colors were chosen from the half contrast hue circle (maximum L cone contrast 4%, maximum S cone contrast 40%), and for “quarter” condition, the half of the half contrast hue circle (maximum L cone contrast 2%, maximum S cone contrast 20%).

### Procedure

Before the recording session started, participants ran several practice trials to familiarize themselves with the task. A trial was initiated by the participant’s button press. Participants were instructed to maintain their fixation at the fixation cross and avoid eye movements or blinks as much as possible during a trial. One trial was 25 s long, during which the pattern flickered at 5 Hz, with smoothly changing hues (this means the full cycle of hues was tested in one trial).

One session consisted of 36 trials. Within a session, all 6 conditions (3 chromatic contrast conditions × 2 directions) were run 6 times each with 6 different starting hue sectors in a random order. During a session, participants could take brief breaks between trials. One session took approximately 20 min. Each participant completed 4 sessions. Thus, each hue sector was measured 48 times at each chromatic contrast (24 trials in counter-clockwise condition, 24 trials in clockwise condition).

A simple Go/No-Go task was given to the participants to ensure that they continuously paid attention to the whole stimulus area. A black “O” or “X” was briefly (50 ms) presented in one of the colored tiles (randomly chosen, see Fig. 2*A*) at the beginnings (with 50 ms jitter) of randomly chosen hue sectors multiple times during a trial. Participants were instructed to press a response button when they detected an O as soon as possible but to ignore when they detected an X. A button press to an O within 900 ms of its onset was considered a hit, and a button press to an X within the same timeframe was considered a false alarm. Performance feedback (hit/false alarm rate) was given to the participant at the end of each trial. There were a total of 36 targets (Os) and 36 distractors (Xs) per hue sector (including all chromatic contrast conditions), spread over 4 sessions.

### E‌EG Recordings and Analysis

EEG was recorded at a sampling rate of 250 Hz from 29 Ag/AgCl electrodes mounted in an elastic cap (EasyCap for BrainAmp, Montage #24) using a BrainAmp MR plus amplifier (Brain Products GmbH, Gilching, Germany). Additional electrodes were attached left, right, and below the participant’s eyes to record eye movements. FCz was used as ground. Right earlobe served as reference during recordings.

Recorded EEG data from 32 channels were analyzed with EEGLAB (Delorme and Makeig 2004) routines and custom-made MATLAB codes. The continuous recording data were dissected into 1-s epochs and detrended. One epoch corresponds to a hue range of 15 deg sector on the stimulus color space. The first epoch of a trial was always discarded to allow SSVEP signal to stabilize. Epochs that contained artifacts such as blinks or saccades were manually rejected from the further analyses. The total rejection rate was 9.0%. After the artifact rejection, the EEG data were re-referenced to the average reference. Epochs of the same hue sectors within the same (chromatic contrast-level and hue-direction) condition were then averaged and appended back in order to reconstruct a continuous 24 s worth of signal. The reconstructed signals were Gabor-transformed (center frequency of Gabor filter was 5 Hz, with a standard deviation of 0.25 Hz, full width at half maximum of ±735.5 ms; this corresponds to approximately ±11 deg in hue space) to compute complex SSVEP amplitudes, from which the magnitude (amplitude) and latency as a function of stimulus hue were computed. SSVEP response amplitude was defined as the absolute value of the complex SSVEP amplitude at 5 Hz. The topographical distribution of SSVEP amplitudes exhibited a clear peak at Iz (Fig. 4), which was chosen for further analyses. The overall amplitude within a participant was normalized to the mean of 1. SSVEP latency was calculated from the phase of the complex SSVEP amplitude. For the target frequency 5 Hz, 2 π phase shift corresponds to 200 ms in latency (see Martinovic and Andersen 2018). Response latency *L* was calculated as

(1) \begin{equation*} L=-\frac{\varphi -\frac{\pi }{2}}{2\pi f}=-\frac{\varphi -\frac{\pi }{2}}{2\pi}\times 200\ \mathrm{ms} \end{equation*}

with phase $\varphi$ (in radians) calculated from the complex amplitudes and frequency *f* = 5 [Hz]. Response phase was shifted by $-\frac{\pi }{2}$ here to account for the phase of stimulation.

95% confidence intervals (CI) for the amplitude and latency data were estimated with 2000 bootstrap samples.

## Results

### Behavioral Data (Go/No-Go Task)

The hue-irrelevant Go/No-Go task was given to the participants to ensure that they equally spread attention to the entire stimulus region rather than a particular spatial area or hue. Therefore, we did not expect a difference in performance between the hue sectors.

**Figure 3:**
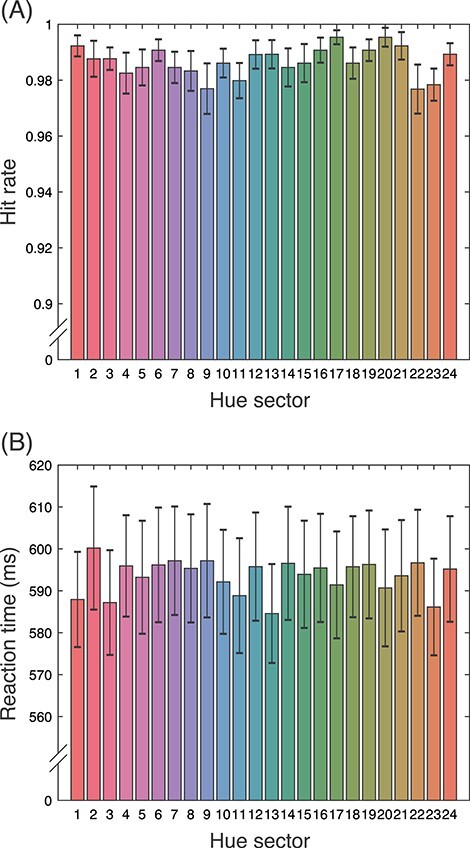
Summary of behavioral data. Mean hit rate (*A*) and reaction time (*B*) of the Go/No-Go task per hue section. Error bar denotes standard error.

Figure 3 shows the average hit rate of the task and the average reaction time to targets. The overall average hit rate is very high (98.8%); in fact, participants hardly made any mistakes (average number of false alarms and misses was 22). This means that they had no trouble performing the task, which is not surprising considering that the targets/distractors are of high contrast and the simplicity of the task itself. On the other hand, the average reaction time is fairly long (591.2 ms), suggesting that although the task was simple and easy enough, it was still reasonably attention-demanding.

Hit rate did not differ across hue sectors (*F*(6.39, 95.85) = 1.26, *P* = 0.28, η^2^ = 0.06; Greenhouse-Geiser adjusted). Analysis of variance on reaction time showed the main effect of test hue (*F*(8.94, 134.04) = 2.66, *P* < 0.01, *η*^2^ = 0.007; Greenhouse-Geiser adjusted), but post hoc multiple comparisons showed no statistically significant differences among any hue combination (Holm’s test, *P* > 0.05).

### E‌EG Data

#### Topographical Distribution and Frequency Analysis

**Figure 4:**
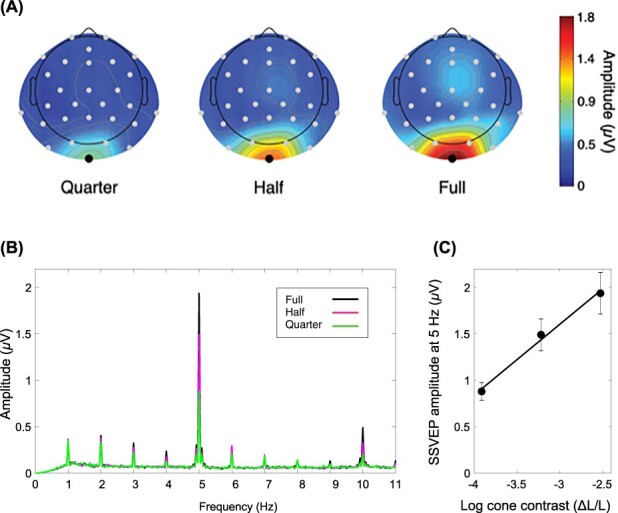
(*A*) Grand mean spline-interpolated iso-contour map of SSVEP amplitude at 5 Hz. Maximal amplitude was located at Iz for all chromatic contrast conditions. (*B*) Mean amplitude spectrum at selected electrode (i.e., Iz). Peaks seen at 1 Hz and its harmonics are due to the Go/No-Go task targets/distractors (see text for details). (*C*) SSVEP amplitude at 5 Hz at 3 log cone contrast level (corresponding to quarter, half, and full conditions from the left), with the fitted linear regression model (solid black line). Error bars denote 1 SE. The positive slope of the line demonstrates that higher chromatic contrast yielded higher amplitude at 5 Hz.

Figure 4 shows the grand mean topographical map of SSVEP amplitude at 5 Hz for the 3 chromatic contrast conditions. As in previous studies using isoluminant chromatic flicker presented foveally (e.g., Andersen et al. 2008; Andersen et al. 2009), the maximal amplitude was located at occipital electrodes, especially at Iz (i.e., inion), for all chromatic contrast conditions. Based on these distribution patterns, Iz was selected for further analyses.

The amplitude spectrum at electrode Iz obtained by Fourier transform of the 24-s reconstructed EEG signal shows pronounced peaks at 5 Hz and 1 Hz with minor peaks at their harmonics. SSVEP amplitudes at 5 Hz were higher for the more saturated colors. We performed linear regression on the SSVEP amplitudes as a function of the stimulus log cone contrast level separately for each participant. The result shows the positive linear relationship between the log cone contrast level and the SSVEP amplitude at 5 Hz [the slope: *t*(15) = 7.35, *P* < 0.01, *d* = 1.84]. The sharp peak at 1 Hz was due to the onsets of the high contrast Go/No-Go task targets and distractors which, except for a small jitter of 50 ms, were synchronized to steps of 1 s. We confirmed this by analyzing the epochs with targets/distractors (half of the epochs) and those without separately; the epochs without targets/distractors did not contain a notable 1 Hz amplitude peak. This additional analysis also showed a negligible difference of 5 Hz amplitude for trials with and without targets/distractors in terms of overall amplitudes and patterns across hues.

#### Hue Dependency of SSVEP Amplitudes

The main question in this study was whether the color coding in the early visual areas such as V1 (which is presumably the primary source of the recorded SSVEP signals) is dominated by cone-opponent signals. If the neurons in such areas receive only L-M cone opponent signals, or only S-cone opponent signals, in other words, if the colors are still represented in cartesian coordinates’ fashion, we would expect the overall signal response profile to form a shape that is line-symmetric with respect to the cardinal axes (see section Latency in Discussion for possible models). Conversely, if the profile was asymmetric to the cardinal axes, this would suggest that the underlying color representation is not just cone-opponent style and likely to include representations that are reconstructed from the cone-opponent signals to form a more complex representation, including intermediate hue representations.

**Figure 5:**
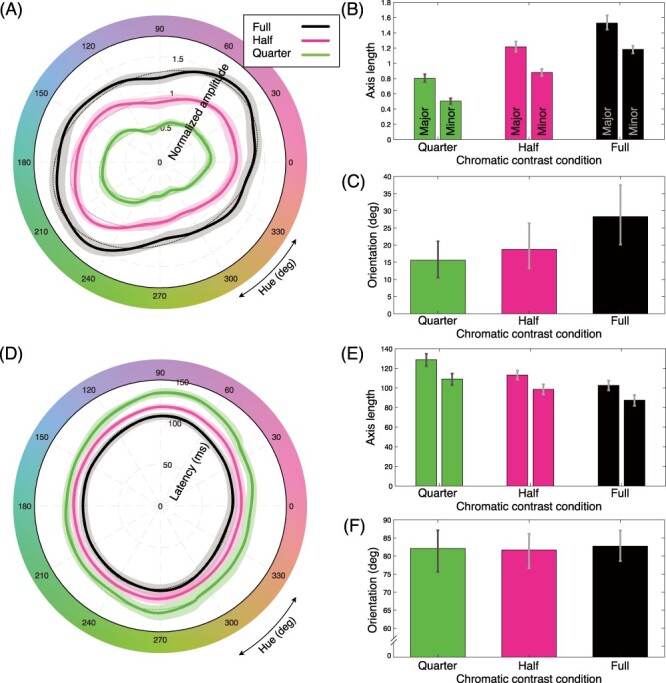
SSVEP Response profiles and parameters of the fitted ellipses. (*A*) SSVEP response amplitude across hues. Solid lines show the mean SSVEP amplitude. (*B*) Lengths of minor and major axes of the best-fit ellipses to the SSVEP amplitude data. (*C*) Orientations of the fitted ellipses. The orientation of zero means that the major axis of the ellipse falls on the L-M axis of the cone opponent space. (*D*) Response latency across hues. Solid lines show the mean latency calculated from the phase of the complex SSVEP response. (*E*) Lengths of minor and major axes of the best-fit ellipses to the response latency data. (*F*) Orientations of the ellipses. Dashed lines in (*A*) and (*D*) are the fitted ellipses to the mean. Shaded areas around the lines (in panels A and D) and error bars (in panels *B*, *C*, *E*, and *F*) show 95% CIs calculated from 2000 bootstrap samples.

Figure 5*A* is a polar plot of the mean SSVEP amplitude across hues. Note that here the SSVEP amplitude was normalized for each participant to a mean of 1 before averaging across participants. It is clear that the more saturated colors elicited stronger SSVEP responses, which indicate the observed response was indeed a chromatic one. Overall shapes of the response show slightly distorted elliptical shapes. There were 2 directions in hue change, but the overall shapes were very similar between these conditions (see Supplementary Materials). To explore the pattern more systematically, we fitted an ellipse to the data for each chromatic contrast condition separately. For the fitting, we used the method described in Szpak et al. (2015). Fitting was done to each bootstrap sample and the mean of the parameters of the fitted ellipses were used for statistical analyses (see Methods). If the cone-opponent hypothesis is true, the orientation of the fitted ellipse would be aligned to the axes to shape the contour symmetric to the cardinal axes. However, for all 3 chromatic contrast conditions, the fitted ellipses were tilted away from cardinal axes rendering the response pattern asymmetric to those axes. For the quantitative comparisons with this hypothesis, we fitted the ellipses to the 3 response curves of SSVEP profiles shown in Figure 5. Best-fitted ellipses are shown with dashed lines in Figure 5*A*. Parameters of the fitted ellipses are shown in Figure 5*B* and *C*. Major axes of the ellipses are significantly longer than minor axes, which indicates that the fitted ellipses are not circles but indeed ellipses. The orientations for the 3 conditions are 15.6 [95% CI: 10.6, 21.1; quarter], 18.8 [95% CI: 13.2, 26.3; half], 28.3 [95% CI: 20.2, 37.5; full] deg; this means that the major axes of the best-fit ellipses stretch in the “lime-magenta” direction. It clearly displays that the major axes deviate from the cone-opponent axes (i.e., vertical and horizontal axes). This distorted shape will be discussed later in detail.

#### Hue Dependency of SSVEP Latencies

One advantage of using the SSVEP technique is that we can calculate the response latency by computing the phase of the complex amplitude of the SSVEP response. We predicted that the response latency would be maximal along S-axis because of the known sluggishness of the S-cone cells (Cottaris and De Valois 1998; also Rabin et al. 1994). We also predicted that the hues with higher chromatic contrast would have shorter latency than those with lower contrast from the previous VEP studies (Rabin et al. 1994; Porciatti and Sartucci 1999).

Figure 5*D* shows the average latency across hues for the 3 chromatic contrast conditions. It is clear that the latencies for more saturated conditions are shorter than less saturated conditions and this is true for all hues around the hue circle. Overall response profiles show that the latencies for hues near S axis tend to be longer than for hues near L-M axis. Major and minor radii are quarter (129 ms, 109 ms), half (113 ms, 99 ms), full (103 ms, 88 ms), and the major axes are longer than the minor by 16 ms on average (Fig. 5*E*). The 3 fitted ellipses were nearly vertical [orientations are 82.1 (95% CI: 75.6, 87.1, quarter], 81.66 (95% CI: 76.6, 86.1; half], 82.7 (95% CI: 78.6, 87.0; full] deg) (Fig. 5*F*).

## Discussion

In the current study, we examined SSVEP response to the various isoluminant colors by sweeping the stimulus hue defined by a cone-opponent color space. We observed higher SSVEP amplitudes and shorter latencies of the SSVEP for higher chromatic contrast. However, SSVEP amplitudes and latencies exhibited different hue-dependencies. SSVEP amplitude significantly differed from what was predicted from the cone-opponent mechanism hypothesis and showed an elliptic profile tilted away from the cardinal axes, with larger responses in the lime-magenta direction. Response latency, on the other hand, was almost symmetric around the S axis with longer latency for S axis hues than for L-M axis hues.

### Amplitude

The main finding in the current study is the SSVEP response amplitude pattern along the hue circle with a tilted elliptic distortion. When the stimulus color smoothly sweeps around the hue circle in the cone-contrast color space, the SSVEP amplitude pattern to these colors is neither aligned with the cardinal axes of this space nor line-symmetric about the cardinal axes. If only cone-opponent mechanisms were underlying the SSVEP, with responses to intermediate hues being the simple summation (either vector or linear) of the cardinal responses, the predicted response profile around the hue circle (Fig. 1) would be symmetric with the cardinal axes being the line of symmetry (Supplementary Fig. 2). The oblique relationship between the hue-dependent magnitude of the SSVEP response and the cone-opponent axes is incompatible with this notion and therefore provides strong evidence that color representations in the sources of the SSVEP result from a nonlinear combination of the cone opponent signals, with intermediate hue representations.

Although the existence of intermediate hue representations in as early as the primary visual cortex has been suggested by physiological studies with primates (Hanazawa et al. 2000; Wachtler et al. 2003), equivalent representations in the human brain have still been a matter of debate. Our data, along with Kuriki et al. (2015), support the notion of such representations in the early visual areas in the human brain, possibly in V1 (for the explanation of the potential source see the later section Source of the SSVEP Signals).

There are also other nontrivial findings in our amplitude data. First, we consistently observed larger responses to the more saturated (i.e., of higher chromatic contrast) colors across hues. Previous chromatic VEP studies found a linear relationship between the VEP amplitude and the log chromatic contrast (Di Russo et al. 2001; Gomes et al. 2006; Souza et al. 2008) and our finding is in agreement with these (Fig. 4*B*). Secondly, for all the colors tested in this study, the SSVEP response amplitude was fairly strong universally across hues. Even for the colors of lowest chromatic contrast (quarter condition), the signal-to-noise ratio was still 5.8 on average, indicating the robust chromatic neural response. The overall strength of the neural response to isoluminant stimuli was the unresolved issue in the preceding fMRI study done by Kuriki et al. (2015), and our current study successfully complemented the finding by demonstrating the sizable response amplitude to our stimuli.

### The Cause of Elongation in Lime-Magenta Direction: Model Comparison

The main question we asked in this study was whether the SSVEP response, presumably from early visual areas, would be dominated by cone-opponent responses as in LGN. The observed tilted elliptic response profile (Fig. 5*A*) strongly indicates that it was not. To quantitatively estimate the possible cause of this tilt, our next question is what kind of model would best describe the obtained chromatic response pattern. We will now answer this question by fitting the potential models to the data and comparing them using Akaike’s information criteria (AIC; Akaike 1974) as a measure of relative model quality.

**Figure 6:**
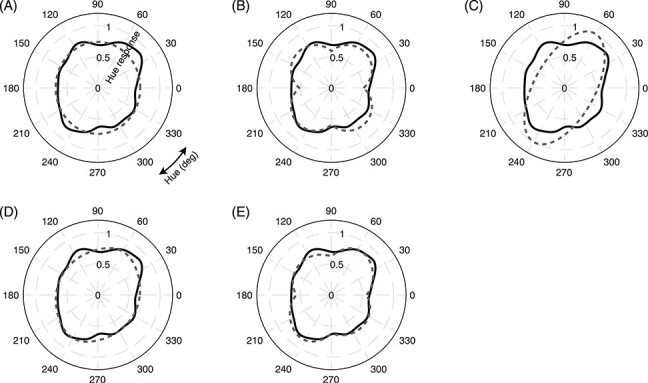
“Purely chromatic” response (difference in SSVEP response amplitude between full and quarter conditions: solid lines) and the best-fit model outputs for 5 candidate models (dashed lines); (*A*) vector-sum cardinal model, (*B*) scalar-sum cardinal model, (*C*) “perceptual” model, (*D*) mixed model (perceptual + vector sum), and (*E*) mixed model (perceptual + scalar-sum). Mixed model (*E*) was chosen as the best model based on its lowest AIC value. See text and Table 1 for further details.

First, for the model comparison purpose, we subtracted the response amplitude of quarter condition from full condition to obtain the “purely chromatic” response. The following is the rationale for this processing. SSVEP amplitudes increased with increasing chromatic contrast (Figs 4 and 5). To assess the changes in SSVEP amplitude as a function of hue, it may be fair to evaluate the changes caused solely by the chromatic contrast. To do so, we need to subtract the SSVEP responses to “zero” chromatic contrast, which should contain responses irrelevant to color processing, from the original responses. However, it is impossible to measure such a response or extrapolate from the observed data without assuming an arbitrary relationship between the SSVEP response amplitude and chromatic contrast changes. Therefore, we decided to evaluate differences in SSVEP amplitude between the smallest and largest chromatic contrast conditions as a close approximation. The black solid line in Figure 6 shows the hue selectivity profile of SSVEP-amplitude differences between the “full” and “quarter” conditions after taking an average across participants. It also exhibits elongation to the first and third quadrant, namely lime-magenta direction. It conversely means that the SSVEP amplitude changes with chromatic contrast changes were smaller in the orange-cyan directions.

Second, we assumed plausible models to account for the shape of this purely chromatic response (for the formulae for the following models, please refer to Supplementary material). The following models are considered; the cardinal axes models (vector-sum and scalar-sum), the perceptual model, the mixed models (weighted sum of cone-opponent and perceptual models) (Fig. 6, dashed curves). Since cone-opponent responses are the form of color signal fed to the cortex, the first possibility is that all the SSVEP responses are comprised of cone-opponent mechanisms: cardinal axes model. The assumption is that the response to any hue along the hue circle is the simple combination of the L-M cells’ response (L-M axis response) and S cells’ response (S-(L + M) axis response). There are 2 possible ways to sum the 2 signals. One is “vector”-sum and the other is “scalar”-sum. The scalar-sum model assumes the linear additivity of electric potentials evoked by the 2 cardinal mechanisms. The response amplitude to a hue angle θ in this scalar model would be the sum of cos(θ) and sin(θ) with a constant relative weight. We considered both models as candidates since these are equally plausible and both models yield symmetric forms across L-M and S- axes, unless any asymmetry in positive and negative components was assumed. The notable difference between the two models is the response amplitudes to the intermediate hues as is seen in Figure 6 (compare dashed curves in panels *A* and *B*).

Another candidate is a perceptual model that is based on the assumption that the SSVEP response amplitude corresponds to the perceived strength/magnitude of the test hue. If the observed data closely resembles the prediction of a perceptual model, it would suggest that the corresponding color representation dominates the source of SSVEP signals, that is, the early visual areas. To implement a “perceptual” value of the hues in this model, we introduced a parameter from The Munsell color system. The Munsell color system was designed to code colors that are equally divided into perceptual differences (Munsell 1905; Nickerson 1940; Newhall et al. 1943; Kuehni 2002), and its measure is more faithful to the color perception than that of CIELAB or CIELUV, which are often used in similar contexts but are insufficient for the purpose of the present study.

The parameter “Chroma” in the Munsell color system stands for the perceptual amplitude of chromatic saturation, and equal Chroma represents an equal difference in vividness with respect to the achromatic point. When we plot 40 Munsell color chips with same “Chroma” (4) of a medium lightness (value = 4) onto a cone-contrast space we used (calculated with spectral reflectance data, available on the University of Eastern Finland webpage [http://www.uef.fi/web/spectral/munsell-colors-matt-spectrofotometer-measured (confirmed on 23 August 2020)], and a flat spectrum illuminant, i.e., EEW), they fall along a nearly perfect ellipse with the longer axis in second and fourth quadrants, roughly perpendicular to the hue selectivity profile of the SSVEP amplitude (Supplementary Fig. 3). This means that among the colors along the locus of the hue circle we tested, which was equally distant in cone-contrast, the Chroma of those colors were not equal across hues; higher values for hues in the first and the third quadrants (i.e., the apparent saturation of colors in these quadrants are perceptually higher than those in other quadrants). This asymmetry might account for the observed asymmetry in SSVEP amplitude data, and therefore we used this model as the perceptual model.

Lastly, we included the mixed models in the evaluation. Each model is the weighted sum of the perceptual model and one of the cardinal models. The relative weight of the perceptual model is a free parameter added to the existing free parameters of each component model.

For the model comparison to decide on the best efficient model of these candidate models, we calculated AIC as follows.

(2) \begin{equation*} \mathrm{AIC}=n{\log}_e\left(\mathrm{RSS}/n\right)+2k, \end{equation*}

where RSS represents the residual sum of squares, *n* represents the number of data points (here, *n* = 6000), and *k* represents the number of parameters for each model (see Table 1). More complex models with more parameters inevitably have better fitting results but also tend to overfit. AIC is one of the information criteria to solve this problem by penalizing the index with the number of parameters. Lower AIC value indicates that the model has a better balance between the 2. We chose the model with the lowest AIC value as the most efficient one.

**Table 1:** Best-fit parameters and AIC of candidate models

	Model 1	Model 2	Model 3	Model 4	Model 5
	Cardinal (vector-su*m*; C1)	Cardinal (scalar-su*m*; C2)	Perceptual (*P*)	Mixed model (ω *P* + (1-ω) C1)	Mixed model (ω *P* + (1-ω) C2)
Number of fitting parameters	2	2	1	4	4
Orientation of longer axis (deg)	0 or 90 (fixed)	0 or 90 (fixed)	61.03 (fixed)	(fixed)	(fixed)
Aspect ratio	1.09	1.14	2.05 (fixed)	1.01 (C1)	1.03 (C2)
Perceptual model weight (ω)	—	—	—	0.54	0.59
Goodness of fit (%)	90.78	91.30	80.72	93.20	94.37
AIC	−32 651.35	−33 346.74	−23 800.42	−36 295.08	−38 564.17

The fitting result is shown in Table 1. The mixed model of perceptual and cardinal (scalar-sum; C2) models (Model 5) showed the best-fit (Fig. 6*E* shows the fitting result). The best-fit weight of the perceptual model was larger (59.1%) than that of the cardinal one, which strongly indicates that the SSVEP response amplitude was dominated by the mechanisms that reflect perceived colors.

That the relative contribution of the perceptual model to the mixed model was large is intuitively reasonable. Since we used subjectively isoluminant stimuli and the detection threshold is nearly equated in S- and L-M axes (Kuriki et al. 2015), the possible difference across hues that could have caused the anisotropy of the SSVEP amplitude profile along the hue circle could be apparent saliency of colors along the hue circle. The mixture of perceptual and cardinal models is also physiologically reasonable given that the single-cell recording studies on the hue selectivity of V1 neurons have shown the mixed population of cone-opponent cells and intermediate hue-selective cells (Lennie et al. 1990; Hanazawa et al. 2000; Wachtler et al. 2003).

### Latency

Our latency data calculated from the response phase show that the latency is longest near S-axis and shortest near L-M axis. This is consistent with the previous psychophysical data showing more sluggish properties of the processing of S-cone isolating stimuli (Wisowaty and Boynton 1980; Ripamonti et al. 2014). The data are also consistent with previous VEP studies (Rabin et al. 1994; Porciatti and Sartucci 1999) as well as a single-cell study of monkey visual cortex (Cottaris and De Valois 1998). Cottaris and De Valois (1998) suggested that the sluggishness of the S-cone color processes originate in primary visual cortex while enhancing the subcortical S-cone signals on the ground that the neurons in LGN that receive S-cone signals are not slow (Gielen et al. 1982; Solomon et al. 1999, 2002). Peak-to-peak latency difference between L/M cone cells and S cone cells in their sample was ~ 19 ms (difference in medians; Cottaris and De Valois 1998). This quantitatively matches the difference we observed in the SSVEP latency between the 2 cardinal directions (16 ms on average).

### Asymmetry Between Lime-Magenta and Orange-Cyan

Our SSVEP amplitude data showed larger responses to hues near “magenta” and “lime” compared with the hues near “cyan” and “orange.” It is interesting to point out that the asymmetry between these pairs of quadrants of the cone-contrast color space has been reported by several studies in the past. For example, Rabin et al. (1994) demonstrated much a larger peak-to-trough VEP amplitude to the hues in the same first and third quadrants of the cone-contrast color space, compared with the hues in the remaining quadrants. For more recent fMRI studies, Goddard et al. (2010) demonstrated that the BOLD response to the modulation in the lime-magenta direction was stronger than to the modulation in the orange-cyan direction, and this bias was observed through V1, V2, V3 and to lesser extent V4 and VO.

On the other hand, in a similar fMRI study with monkey subjects, Lafer-Sousa et al. (2012) discovered the asymmetry in the opposite direction, that is, stronger response to the orange-cyan direction. No clear explanation was given so far to the discrepancy between the 2 studies except for the obvious difference in subject species, which seems to be supported by single-unit studies demonstrating the bias in orange-cyan direction (Conway 2001; Solomon and Lennie 2005). Greater “Chroma” values in the Munsell system, which is based on human perception, in lime-magenta direction contributed to the tilted SSVEP response shape in our model (see section Latency), and possibly to Goddard et al.’s. (2010) data. Perhaps, macaques have different isochroma contour from that of humans which would have resulted in the apparently opposite fMRI results.

The asymmetry between the lime-magenta and orange-cyan has been also found by psychophysical studies (Krauskopf and Gegenfurtner 1992; Witzel and Gegenfurtner 2013). Hue discrimination performance is better along orange-cyan direction than along lime-magenta (Witzel and Gegenfurtner 2013), whereas saturation discrimination performance appears to be better along lime-magenta than along orange-cyan (Krauskopf and Gegenfurtner 1992). Since the flicker of our stimulus was in the saturation direction (alternations between the test hue and the background gray), the larger amplitude in the lime-magenta direction in our data can be interpreted as more efficient representation of saturation in the direction, which may correspond to the aforementioned psychophysical data.

Unfortunately, we do not have a reasonable explanation of the opposite bias reported by previous studies or to the origin of the asymmetry in the cone-contrast color space. However, we do believe that the asymmetry we observed in our SSVEP data is closely related to the perceptual values of the colors, as mentioned in the previous section.

### Source of the SSVEP Signals

Electrophysiological signals are inherently ambiguous as to their sources, which sometimes make it difficult to interpret the results of EEG or SSVEP studies. In the current study, we argue that the observed SSVEP response pattern to various hues mainly reflects the representation of early visual areas, especially V1, based on the following reasons. Firstly, previous studies that combined EEG and fMRI recordings pinpointed the main signal source of EEG signals to the flickering stimuli as the primary visual cortex (Di Russo et al. 2007). Secondly, the observed topographical distribution of the SSVEP signals was clearly localized in the occipital channels, especially Iz. Although the topographical peak and the location of the signal source does not necessarily correspond to each other, our topography matches the ones observed by previous studies using foveal isoluminant flickers, which also performed source localization with much denser electrode mappings and localized the source as early visual areas (V1–3) (Müller et al. 2006; Andersen et al. 2008; Andersen et al. 2009; Andersen et al. 2012). Please note that we do not claim that the SSVEP is exclusively elicited by a single source: as demonstrated in Andersen et al. (2012), it is possible to reliably extract a later bi-lateral parieto-occipital source which most likely corresponds to MT/LOC (see also Störmer et al. 2013). However, this later source is not reflected in the electrodes analyzed in our current study (please see Andersen et al. 2012 for further details; note that although that paper employs luminance flicker, the exact same sources can be extracted using isoluminant flicker as we have confirmed through reanalysis of multiple datasets in our lab). Lastly, the average signal latency calculated from the phase of the response ranged from 90 to 130 ms. This corresponds to the response latency values of the neural latency to the isoluminant colors in the macaque primary visual cortex (Cottaris and De Valois 1998).

### Potential Effect of Nonisoluminant Components in the Stimulus

Although we designed our checkerboard pattern to elicit purely chromatic SSVEP response with isoluminant hues as flickering target stimulus, it is possible that the responses may have been affected by nonisoluminant components of the stimulus. These potential effects can be from the nonflickering dark edges of the checkerboard pattern and the potential residual luminance artifact contained in the test hues. We briefly discuss the potential effects of these in this section.

#### Black Edges

Xing et al. (2015) showed the VEP to the isoluminant colors weakened with a thin luminance edge. It is therefore possible that overall response amplitude could have been larger in our data if we removed the black grid from our stimulus. However, the grid was present during any hue presentation, and there is no reason to believe that this distorted the response pattern in any specific way and would change the conclusions.

#### Residual Luminance

As it has been shown by the phase analysis, the deviation of hue angle of S-cone selective stimulation was ~ 5 degree from 90–270 degree (i.e., S-cone selective on calculation) axis, because the S-cone systems are known to be irrelevant for the luminance-based system (Eisner and MacLeod 1980; Stockman and Sharpe 1999). Hence, it has been confirmed that the hue direction around 90–270 deg was subjectively isoluminant. We estimated the luminance channel response as the weighted sums of L- and M-cone responses (*L* + ϕ*M*), and the relative M-cone weight ϕ was determined by the method of flicker photometry (see Methods; Ahn and MacLeod 1993; Kuriki and MacLeod 1998). If any deviation from isoluminance takes place, it means the deviation of ϕ from the actual value, and that would appear in the form that the effect of deviation from isoluminance becomes larger in proportion to the distance from the S-cone axis. Given that any deviation from theoretical isoluminance is present, it would appear in a symmetric manner with respect to the 90–270 deg (S) axis. Therefore, even if there had been any deviation from the purely isoluminant plane in our color stimuli, that would not have caused the SSVEP amplitude profile asymmetric to the cardinal axes and hence would not affect our conclusion.

### Potential Effect of Progressive Adaptation

During the 25 s of stimulus presentation, there may have been a progressive adaptation to colors and this adaptation may have affected the response pattern (for the same argument, please see Kuriki et al. 2015). However, we believe that the effect of progressive adaptation was small, if any, and did not affect our result in the way that would change our conclusion for the following reasons.

Firstly, if adaptation to the hue sequences had happened, this adaptation would have been in opposite direction between counterclockwise sweep and clockwise sweeps. This would have therefore resulted in a symmetric shift in response pattern between the 2 conditions. In the observed data, however, the difference between the 2 sweep conditions was negligible as seen in Supplementary Figure 1.

Secondly, participants experienced both clockwise and counterclockwise sweeps starting from 24 possible hue sectors, presented in a pseudo-random order, each with an equal number of trials. This means that all the hues (and all the contrast levels) were equally stimulated over time, and therefore the effect of adaptation should have been also equal across conditions and hues.

Lastly, a previous fMRI study using similar color-sweeping stimuli ran an experiment to see if adaptation to such stimuli changes the color appearance and showed no significant effects (Kuriki et al. 2015). Since our stimulus was essentially the same as the one used by them, we could justify our extrapolating their control experiment to ours.

### Limitations of the Current Study and Future Directions

In this last section, we would like to mention the limitations and the future directions of this study. Although our data successfully demonstrated robust hue selective responses from the early visual areas, some of the details of the hue representation are yet to be explored. One of such is the tuning width of the assumed hue-selective neurons in the areas. Some studies suggested that the multiple narrowly tuned hue channels represent the perceived hues in a similar fashion as in the orientation representations (Klauke and Wachtler 2015; Emery et al. 2017). Emery et al. (2017), for example, suggested 7 to 8 narrow channels underlying hue perception based on psychophysical data. Our current study was not designed to reveal these natures of the presumed channels/neurons. We did however run a pilot study employing the same technique as here to tackle this question. We employed the same experimental paradigm as Peterzell and Norcia (1997), which explored the numbers and tuning properties of the spatial frequency channels using the masking paradigm. Following this study, we explored the SSVEP response to the same isoluminant colors in the simultaneous presence of the masker color (intermediate hues with higher chromatic contrast than the test hues; Kaneko et al. 2019, conference abstract). In this study, the same isoluminant checkerboard was used but now half of the tiles were of masker color, whereas the other half was filled with test hues. The results showed the selective reduction of SSVEP response amplitude at the masker hues, suggesting the existence of the narrowly tuned hue-selective channels at those intermediate hues.

We also would like to briefly mention the individual differences. Throughout this paper, we discuss the grand mean of the SSVEP responses, focusing on the hue selective response features universally seen across our participants. However, we also noticed the large individual differences in the SSVEP response amplitudes, in terms of overall amplitude as well as of skew in hue selectivity. At this moment, it is not possible to confirm the cause of such differences in the data. We can speculate that the unique response profile of a participant is closely related to their perception, and anticipate to find a correlation between the SSVEP responses and behavioral data (such as detection threshold, visibility matching, unique hues, etc.). Our future study will explore these potential causes of individual differences.

## Funding

Japan Society for the Promotion of Science (JSPS KAKENHI grant number JP18K13365 to S.K., JP18H04995 to I.K.)

## Notes

S.K. was also supported by the grant from Building of Consortia for the Development of Human Resources in Science and Technology program by Japan Science and Technology Agency. S.K.A. is very grateful to Satoshi Shioiri for inviting him to Tohoku University, which enabled this collaboration. Part of this study has appeared in the form of conference proceedings (Kaneko et al. 2018). Data and codes used in this study are available at https://osf.io/m47df/.

## Supplementary Material

SupplementaryMaterials_KanekoKurikiAndersen_final_tgaa059

HueSel_demo_movie2_tgaa059
