# Supplementary material for: Steady-State Visual Evoked Potentials Elicited from Early Visual Cortex Reflect Both Perceptual Color Space and Cone-Opponent Mechanisms
Source: Cereb Cortex Commun. 2020 Sep 1;1(1):tgaa059. doi: 10.1093/texcom/tgaa059 (PMC8152915; doi:10.1093/texcom/tgaa059)
Supplement: SupplementaryMaterials_KanekoKurikiAndersen_final_tgaa059 [file supplementarymaterials_kanekokurikiandersen_final_tgaa059.docx]

**Supplementary Materials**

Steady-state visual evoked potentials elicited from early visual cortex reflect both perceptual color space and cone-opponent mechanisms

**Sae Kaneko, Ichiro Kuriki, Søren K. Andersen**

Contents:

**Figure S1.** Grand mean of normalized SSVEP amplitude and latency shown separately for each sweep direction.

**Figure S2.** Predictions of SSVEP amplitude based on cardinal hypotheses.

**Figure S3.** Iso-Chroma colors in the Munsell system plotted in a cone-opponent color space.

**Models.** Formulae for the numerical model fitting.

Other (not included in this document):

**Movie S1.** A movie of a trial. This movie is for demonstration purpose only. Color or presentation timing of the stimulus may not be accurate due to the rendering process of this movie.

**Figure S1.** Grand mean of normalized SSVEP amplitude (A) and latency (B), separately shown for each sweep direction (counterclockwise, CCW and clockwise, CW)

**Figure S2.** Predictions of SSVEP amplitude based on cardinal hypotheses (red lines), vector-sum prediction (A) and linear-sum prediction (B). Either way of summation would result in the symmetric response profile along the cardinal axes. Aspect ratios in these predictions are arbitrary.

**Figure S3.** Iso-Chroma colors in the Munsell color space plotted in cone-opponent color space. The horizontal and vertical axes are the same as Figure 1 in the main text. 40 Munsell color chips with same *Chroma* (= 4) of a medium lightness (*Value* = 4) are plotted here. They were derived by using a spectral reflectance data measured by University of Eastern Finland with a flat spectrum illuminant, i.e., equal energy white (EEW). EEW was used as a background gray of our stimuli and is the origin of this figure. The fitted ellipse is centered at (0.0, 6.0 x 10^-5^) and the tilt angle of the major axis is 151.0 deg. The aspect ratio of the ellipse *on this figure* is 2.05. The goodness-of-fit of the ellipse, based on sum-of-squared error, is 98.5%.

**Models**. Formulae for the numerical model fitting. Each model parameter was optimized by minimizing squared error from the observed value. See main text for details.

| Model 1. Cardinal-axis (vector sum) model: *C1*   | S1 |
| --- | --- |
| Model 2. Cardinal-axis (linear sum) model: *C2* ** | S2 |
| Model 3. Perceptual (Munsell-chip-based) model: *P* *θ_tilt_* = 61.0 (deg) …derived by fitting an ellipse to Munsell chip chromaticity (fixed) *r*_a_ / *r*_b_ = 2.05 … aspect ratio (fixed)  | S3 |
| Model 4. Combined model: ω*P* + (1 - ω) *C1* Parameters for *x*_est1_’, *y*_est1_’ (*A_C1,LM_, A_C1,S_*) and *x*_est3_’, *y*_est3_’ (*A_C3_*) are also optimized together with the relative weight ω by minimizing squared error *ε_sq_*_4_.  | S4 |
| Model 5. Combined model: ω*P* + (1 - ω) *C2* Parameters for *x*_est2_’, *y*_est2_’ (*A_C2,LM_, A_C2,S_*) and *x*_est3_’, *y*_est3_’ (*A_C3_*) are also optimized together with the relative weight ω by minimizing squared error *ε*_sq5_.  | S5 |
